# Phytomedicines modulate pyroptosis in cardiovascular diseases: pre-clinical evidence, limitations and translational perspectives

**DOI:** 10.3389/fphar.2026.1885508

**Published:** 2026-09-10

**Authors:** Zichen Zhu, Huici Zhu, Zhuoyue Zhang, Jiaying Hu, Baonian Liu, Haidong Guo

**Affiliations:** Department of Anatomy, School of Integrative Medicine, Shanghai University of Traditional Chinese Medicine, Shanghai, China

**Keywords:** cardiovascular diseases, multi-botanical botanical formulations, plant-derived metabolites, pyroptosis, therapeutic strategies

## Abstract

Cardiovascular diseases (CVDs) represent the leading global cause of disability and premature mortality, imposing an immense burden on global public health and medical systems. Notwithstanding the availability of multiple clinical drugs, controlling the incidence and mortality of CVDs remains a formidable challenge. Pyroptosis, a specialized pro-inflammatory programmed cell death marked by membrane pore formation, is involved in the pathogenic mechanisms of several CVDs. Phytomedicines, encompassing plant-derived metabolites and multi-botanical botanical formulations, can inhibit pyroptosis by regulating related pathways, thereby exerting cardiovascular protective effects. This review elaborates on the core molecular characteristics, morphological changes, and canonical/non-canonical activation mechanisms of pyroptosis. Systematically delineate the pivotal function of pyroptosis in the pathogenic advancement of predominant CVDs. Additionally, analyze the regulatory impacts and fundamental molecular processes of natural products and multi-botanical botanical formulations on pyroptosis in various CVDs. Collectively, this research positions the manuscript as a systematic preclinical review focused on pyroptosis-targeted natural therapeutic candidates, systematically outlines pyroptosis-mediated pathological cascades in CVDs, and highlights the translational potential of natural agents that block pyroptosis. Nearly all supporting evidence summarized herein remains pre-clinical; well-designed human studies are urgently required before any real-world clinical application can be considered.

## 1 Introduction

Cardiovascular diseases (CVDs) rank as the leading global cause of premature death and disability, with rising prevalence driven by population aging and imposing heavy medical and economic burdens worldwide. Conventional clinical drugs cannot effectively curb CVD incidence and mortality (Joseph et al., 2017). They impose a heavy global medical burden, and abundant preclinical evidence has validated multi-target cardioprotective effects of botanical preparations via modulating diverse pathological cascades such as inflammation, oxidative stress and regulated cell death (Dai et al., 2024).

Pyroptosis, an inflammation-associated form of programmed cell death, participates in the pathological progression of diverse cardiovascular disorders (North and Sinclair, 2012). In recent years, pyroptosis-regulating effects of botanical agents have drawn extensive attention. Plant-derived active metabolites exist across global medicinal flora rather than being limited to Chinese materia medica, while multi-botanical formulas exert multi-target anti-cardiac injury via inhibiting pyroptosis.

This review systematically summarizes pyroptosis-mediated cardiac damage across distinct CVD subtypes and elaborates multi-molecular regulatory mechanisms of single phytochemicals and compound prescriptions, aiming to lay a theoretical foundation for translational development of natural cardiovascular therapeutic candidates.

## 2 Pyroptosis

**FIGURE 1:**
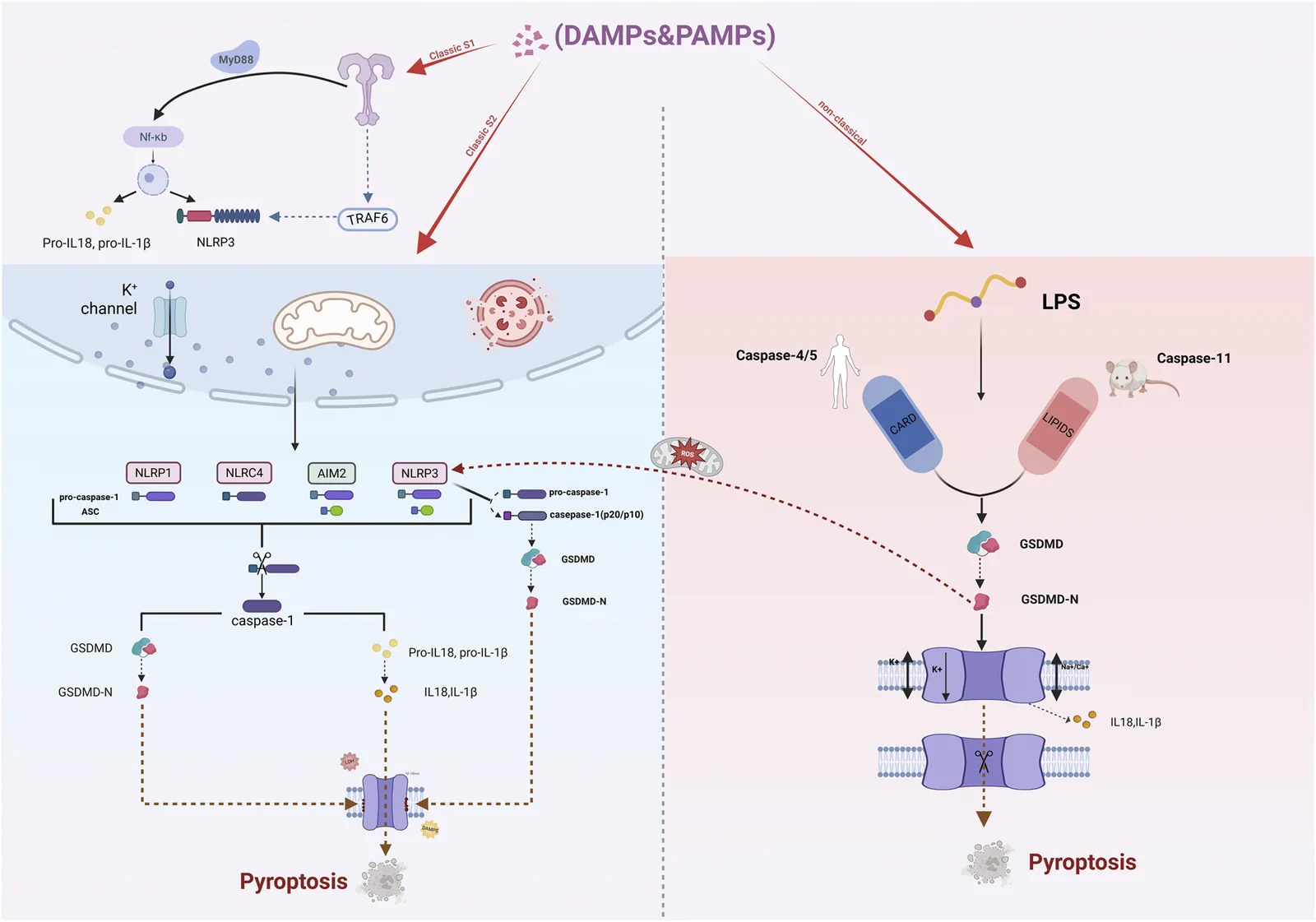
Pyroptosis is a pro-inflammatory programmed cell death, proceeding in three stages (initiation: transcription of inflammation-related molecules. Effector: inflammatory caspases cleave GSDMD to form membrane pores. Lysis: cell rupture and release of inflammatory factors) and mediated by two pathways. The canonical pathway relies on dual signals to activate NLRP3 inflammasome and caspase-1, while the non-canonical pathway is triggered by intracellular LPS activating caspase-4/5 (human) or caspase-11 (mouse). Both require GSDMD to induce membrane pore formation.

Pyroptosis is an inflammatory programmed cell death distinct from apoptosis and necrosis. Discovered in 1992 and formally named in 2001, it exerts dual physiological and pathological functions, with its core cascades critical to cardiovascular inflammatory injury (Galluzzi et al., 2018) (Figure 1).

### 2.1 Molecular characteristics and morphological changes of pyroptosis

Pyroptosis proceeds via three sequential functional phases. The initiation stage activates transcription of inflammatory mediators without obvious cellular morphological deformation (Lu et al., 2014). In the effector phase, inflammatory caspases cleave full-length GSDMD to produce lipophilic GSDMD-NT fragments, which oligomerize and embed into cell membranes to form 10–16 nm transmembrane pores, enabling bidirectional leakage of ions, ATP and immature cytokines (Wei et al., 2024). Continuous pore expansion ultimately induces cell swelling and lysis, releasing LDH and mature pro-inflammatory cytokines (Galluzzi et al., 2018). All pyroptotic phenotypes rely on gasdermin family cleavage and are categorized into canonical and non-canonical activation axes, both widely detected in cardiovascular tissue under pathological stress.

### 2.2 Molecular mechanism and regulatory network of the canonical pyroptosis pathway

The canonical two-signal pathway is the most extensively characterized pyroptosis cascade in atherosclerosis, myocardial infarction and other heart disorders. The priming signal engages TLR-NF-κB signaling to upregulate transcription of NLRP3, pro-IL-1β and pro-IL-18; TRAF6-mediated non-transcriptional NLRP3 ubiquitination further facilitates inflammasome assembly (Xing et al., 2017). Cellular stress stimuli including K^+^ efflux, mitochondrial dysfunction and lysosomal rupture constitute the secondary activating signal, inducing NLRP3 conformational rearrangement, ASC recruitment and autocleavage of pro-caspase-1 (Zhou et al., 2011). Beyond NLRP3, AIM2 and NLRC4 inflammasomes also activate caspase-1 to process GSDMD and mature inflammatory cytokines (Martinon et al., 2009; Lamkanfi and Dixit, 2012; Wei et al., 2024).

### 2.3 Non-canonical pyroptosis pathway

The non-canonical inflammasome-independent cascade is triggered by intracellular LPS with species-specific ligand recognition patterns in humans and rodents (Kayagaki et al., 2011; 2013; Hagar et al., 2013; Shi et al., 2014). Interferon-induced GBP proteins promote cytoplasmic LPS exposure to strengthen caspase binding efficiency (Santos et al., 2018; 2020). Activated caspase-4/5/11 cleaves GSDMD, and the resulting N-terminal fragments target mitochondria to generate ROS and mtDNA, which secondarily amplify NLRP3 and AIM2 inflammasome activity and aggravate cardiac inflammatory lesions (Liu et al., 2016). Endothelial cells primarily rely on this non-canonical cascade under metabolic and hemodynamic stress in CVDs.

### 2.4 Other gasdermin family members in cardiovascular pyroptosis

Apart from GSDMD, GSDME and GSDMA participate in pyroptotic injury within cardiac tissue. All full-length gasdermin proteins carry autoinhibitory C-terminal domains, and proteolytic cleavage releases pore-forming N-terminal functional segments (Liu et al., 2016). GSDME is specifically cleaved downstream of the Bnip3-mitochondrial-caspase-3 cascade, acting as the core executor of doxorubicin-triggered cardiomyocyte damage (Zheng et al., 2020; Xue et al., 2025; Wei et al., 2023; Yang et al., 2025). In contrast, human GSDMA lacks conserved caspase cleavage sites; its only verified cleavage mechanism relies on streptococcal SpeB protease, and few cardiac knockout studies support its functional relevance to cardiovascular pyroptosis (Tanaka et al., 2013).

## 3 Pyroptosis in cardiovascular diseases

**FIGURE 2:**
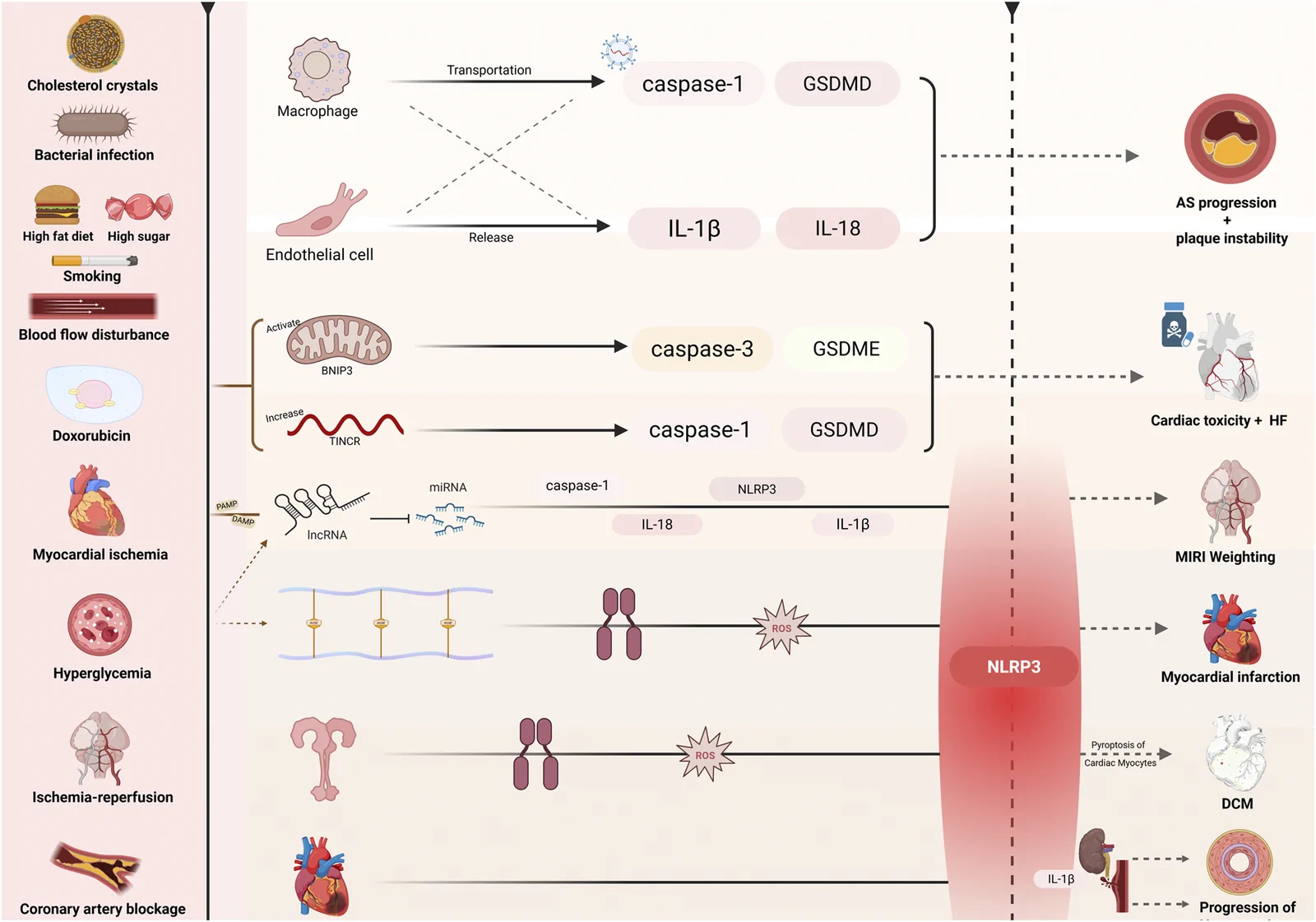
Pyroptosis is widely involved in the pathological processes of CVDs like AS, MI, MIRI, and DCM. It mainly activates inflammasomes and inflammatory caspases, induces cell lysis, and releases pro-inflammatory factors, thereby exacerbating cell damage, inflammation amplification, and disease progression—for example, promoting plaque formation in atherosclerosis and impairing cardiac function in MI.

Moderate pyroptosis maintains cardiac tissue homeostasis, whereas excessive activation drives inflammatory injury across nearly all cardiovascular pathologies (Man et al., 2017). Distinct cell subsets exhibit divergent pyroptotic signaling profiles that determine disease-specific inflammatory phenotypes, laying a theoretical basis for targeted intervention via phytomedicines (Badimon et al., 2019; Ji et al., 2021) (Figure 2). Endothelial cells prefer caspase-4/5 non-canonical cascades triggered by metabolic or mechanical stress; macrophages predominantly rely on NLRP3 inflammasome activation, with interstitial macrophages in heart failure additionally engaging NLRC4/AIM2; cardiomyocytes activate dual NLRP3/GSDME signaling in acute ischemia but shift toward GSDME-dominant pathways under chronic metabolic or toxic stimuli; cardiac fibroblasts universally undergo GSDME-mediated cleavage to induce interstitial fibrosis, while vascular smooth muscle NLRP3 activation is unique to hypertensive remodeling (Ji et al., 2021).

### 3.1 Lipid-driven chronic vascular injury

Atherosclerosis represents lipid-induced chronic inflammatory vascular pathology prone to plaque rupture and fatal cardiovascular events (Tsao et al., 2023; Moriya, 2019; Xu et al., 2018). Dyslipidemia, sustained hyperglycemia and disturbed shear stress initiate non-canonical pyroptosis in endothelial cells, with toxic pollutants and nicotine further amplifying this process. Accumulated lipid crystals trigger robust NLRP3 inflammasome assembly in monocyte-derived foam cells to promote IL-1β secretion and necrotic core expansion, an effect exacerbated by bacterial infection and hematopoietic gene variants (Duewell et al., 2010; Liu et al., 2020). Late-stage perivascular cells undergo GSDME-dependent pyroptosis, and exosomal cargo mediates intercellular transmission of pyroptotic signals within vascular lesions (Tsuchiya, 2020). Notably, most current mechanistic data originates exclusively from ApoE^−/−^ rodent models, lacking validation using human atherosclerotic plaque specimens and clinical hemodynamic data.

### 3.2 Acute myocardial ischemia and reperfusion damage

Persistent coronary occlusion induces acute myocardial infarction, while subsequent reperfusion triggers secondary oxidative cardiac injury, both relying on coordinated pyroptotic activation across multiple cardiac cell populations (Ibanez et al., 2018; Bugger and Pfeil, 2020). Hypoxic stress coactivates cardiomyocyte NLRP3 and caspase-3/GSDME signaling to deteriorate cardiac contractile function and elevate long-term heart failure risk (Toldo and Abbate, 2018; Wang et al., 2023; Abbate et al., 2020). Fibroblastdothelial cells activate caspase-4 to disrupt microvascular barrier integrity, infiltrating macrophages boost NLRP3-dependent inflammation, and cardiac fibroblast GSDME cleavage drives irreversible ventricular remodeling (Hou et al., 2022). Mitochondrial ROS accumulation forms a positive feedback loop amplifying inflammasome activation via endothelial and macrophage crosstalk, with fibroblast pyroptosis further exacerbating interstitial fibrosis (Tang et al., 2019). Limitations of existing preclinical models include reliance on permanent coronary ligation without clinical revascularization simulation and single-time-point detection without long-term dynamic pyroptosis tracking.

### 3.3 Metabolic and drug-induced cardiomyopathy

This section integrates diabetic cardiomyopathy and doxorubicin cardiotoxicity, two chronic myocardial injuries driven by distinct toxic stimuli. Diabetic cardiomyopathy develops independent of coronary stenosis under chronic glucolipotoxicity (Tan et al., 2020). Chronic high glucose upregulates NLRP3 and GSDME expression via non-coding RNA regulatory axes, whereas Nrf2/HO-1 signaling counteracts ROS-mediated pyroptosis (Liu et al., 2024; Wei et al., 2023). Lipotoxic stimuli trigger endothelial non-canonical pyroptosis and fibroblast GSDME cleavage via exosomal signal transmission (Liu et al., 2024; Cai et al., 25). Most *in vitro* diabetic models only apply single high-glucose culture without simulating clinical combined hyperlipidemia. By contrast, anthracycline doxorubicin causes dose-dependent cardiac adverse effects in 25% of treated patients (Lefrak et al., 1973). Its cardiotoxicity is predominantly mediated by Bnip3-caspase-3-GSDME cascades with minor NLRP3 inflammasome involvement; the lncRNA TINCR further exacerbates inflammasome transcription (Meng et al., 2019; Zheng et al., 2020). Endothelial caspase-4 and macrophage NLRP3 activation jointly aggravate myocardial inflammatory fibrosis (Xue et al., 2025). Relevant preclinical research commonly adopts drug dosages far exceeding human clinical equivalents, while studies investigating low-dose long-term chemotherapy cardiac damage remain scarce.

### 3.4 Chronic cardiac remodeling (heart failure & hypertension) and autoimmune-associated myocardial injury

Sustained pyroptotic signaling mediates irreversible cardiac remodeling in heart failure and hypertensive heart disease, while inflammatory myocardial lesions can also arise secondary to autoimmune arthritis. Pressure overload and Ang II stimulation trigger vascular smooth muscle-specific NLRP3 activation, endothelial caspase-4 barrier disruption, and cardiomyocyte/fibroblast GSDME-mediated hypertrophy and fibrosis, forming a self-amplifying pressure-inflammation circuit (De Miguel et al., 2021). In terminal heart failure, persistent mechanical stress simultaneously activates cardiomyocyte NLRP3 and GSDME and impairs endothelial microcirculation and induces multi-inflammasome activation in interstitial macrophages; fibroblast GSDME cleavage results in permanent myocardial scarring (Toldo et al., 2022; Hou et al., 2022). Current research rarely distinguishes preserved versus reduced ejection fraction heart failure subtypes for pyroptosis mechanistic comparison. Beyond primary cardiac disorders, adjuvant arthritis provokes systemic inflammation that drives myocardial pyroptosis via the lncRNA/miR-21/TLR4/NLRP3 axis, leading to elevated circulating inflammatory cytokines and myocardial LDH leakage (Fu et al., 2024).

## 4 Regulation of cardiovascular diseases by phytomedicines through modulating pyroptosis

**FIGURE 3:**
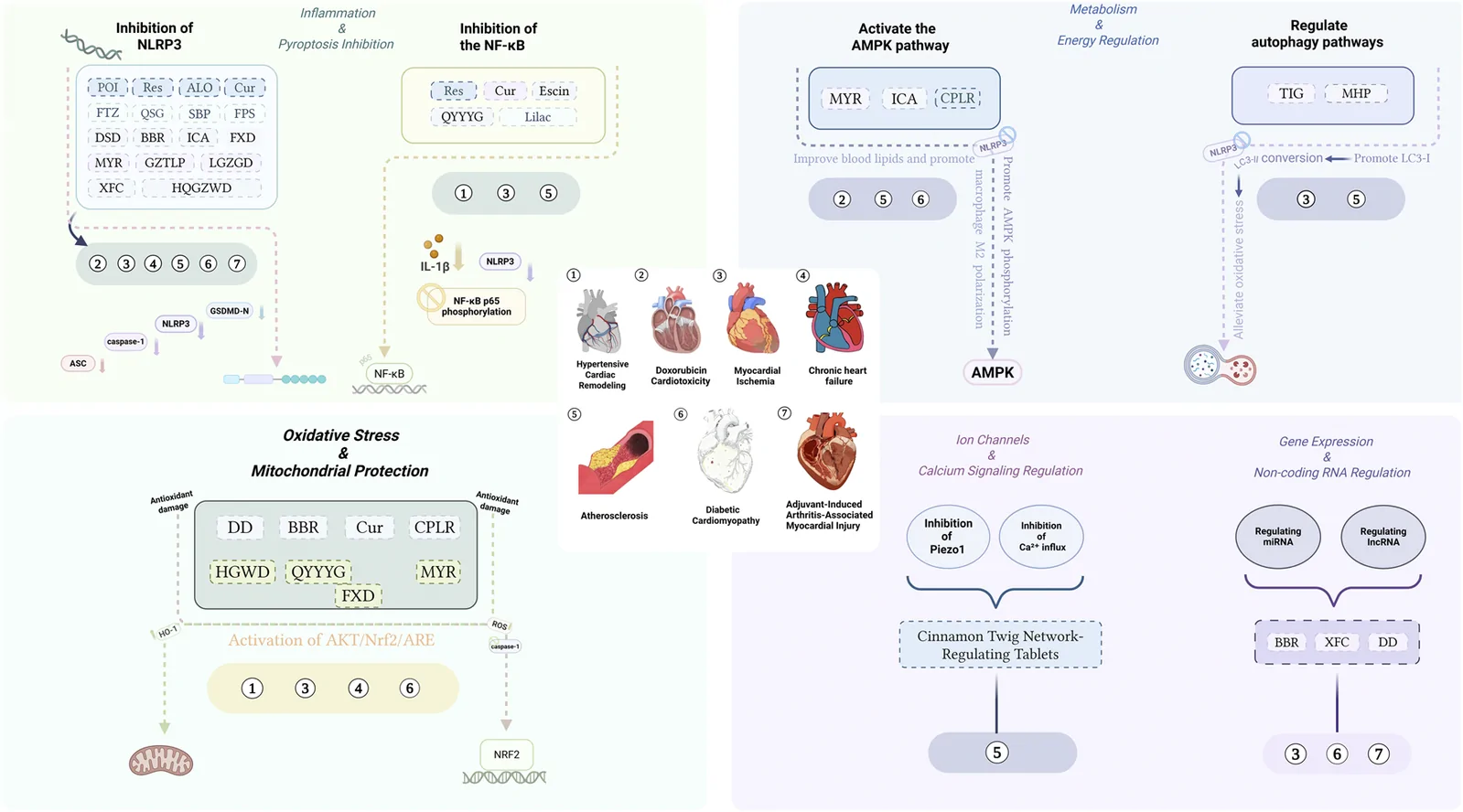
Natural products (single plant-derived metabolites and multi-botanical botanical formulations) exert cardiovascular protective effects by regulating pyroptosis. Single plant-derived metabolites such as polydatin, resveratrol, and berberine inhibit pyroptosis by targeting pathways like NLRP3, NF-κB, or activating Nrf2/AMPK. Multi-botanical botanical formulations, including Fufang Zhenzhu Tiaozhi, Shexiang Baoxin Pill, and Huangqi Guizhi Wuwu Decoction, also suppress pyroptosis, thus alleviating CVD-related damage.

Many plant-derived metabolites and multi-botanical botanical formulations deliver distinct preventive or therapeutic anti-pyroptotic activity against cardiovascular disorders. A growing number of Research indicates that single plant-derived metabolites exhibit considerable preventive effects in CVDs via modulating essential pathways, including inflammation, oxidative stress, lipid metabolism, and programmed cell death (Zhang X. et al., 2023; Xu et al., 2025a). In addition, single plant-derived metabolites demonstrate multifaceted and multi-targeted synergistic effects in cardiovascular protection, providing a unique strategy for the intervention of complex CVDs. On the other hand, multi-botanical botanical formulations, distinguished by their multi-faceted and multi-target synergistic effects, also show unique advantages in intervening in the pathological process of CVDs mediated by pyroptosis (Dai et al., 2024). We concentrate on examining the potential regulatory mechanisms of typical single plant-derived metabolites and multi-botanical botanical formulations in influencing the pathological process of CVDs by regulating pyroptosis (Figure 3) (Table 1).

**TABLE 1:** The Mechanisms of Action of Metabolites in Different Cardiovascular Diseases through pyroptosis.

Agent (Abbreviation)	Agent class	Extraction/Preparation type	Evidence tier	*In vitro* parameters	*In vivo* parameters	Control groups	Main therapeutic outcomes	Core regulated pathways	References
Polydatin	Purified monomer	Purified chemical compound	T3	No *in vitro* experiment conducted	ApoE^−/−^ and C57BL/6J mice, oral gavage, HFD feeding for 12 weeks, single therapeutic dose, 12 weeks	Blank vehicle control	↓Aortic plaque area and lipid deposition, ↑plaque stability, ↓pyroptosis-related protein expression and IL-1β/IL-18 release	NLRP3/mTOR/autophagy axis	Zhang et al. (2023a)
Resveratrol (Res)	Purified monomer	Purified chemical compound	T2+T3	VSMC calcification model, 10 mmol/L, 10 mmol/L, 7–10 days	ApoE^−/−^ mice, intraperitoneal injection 5 mg/kg, last 3 weeks of 16-week HFD feeding, single dose, 16 weeks	Blank control + PC (parthenolide)	↓VSMC calcification, inflammation and pyroptosis	NF-κB/NLRP3/IL-1β signaling	Li et al. (2024c)
Aloperine (ALO)	Purified monomer	Purified chemical compound	T2+T3	Ox-LDL induced THP-1 macrophage model, 12.5/25/50 μM, 12.5 μM, 24 h	ApoE^−/−^ mice, oral gavage 40 mg/kg/d, single dose, 4 weeks	Blank control + V (DMSO)	↓Aortic plaque formation, IL-1β secretion and caspase-1 activity	p38/JNK/NLRP3 inflammasome	Wang et al. (2025)
FTZ (compound Pearl Modulation)	Botanical formula	Aqueous granule	T4+T3	No *in vitro* experiment conducted	ApoE^−/−^ mice, oral gavage 1.2 g/kg/d, 1.2 g/kg, 14 weeks	Blank control + PC (simvastatin)	↓Blood lipid levels, aortic plaque area and macrophage pyroptosis	AMPK/NLRP3/mitoROS pathway	Shao et al. (2024)
Guizhi Tongluo tablets (GZTLT)	Botanical formula	Aqueous tablet	T4+T3	No *in vitro* experiment conducted	ApoE^−/−^ mice, oral gavage 0.52–2.08 g/kg, 0.52 g/kg, 8 weeks	Blank vehicle control	↓Blood lipid levels and foam cell formation	Piezo1/Ca^2+^/NLRP3/GSDMD axis	Pan et al. (2024)
Syringa oblata Lindl (SO)	Crude extract	Ethanol extract	T2+T3	LPS + ATP induced RAW264.7 macrophage model, 1.25–10 μg/mL, 1.25 μg/mL, 24 h	LAD-ligated AMI mice, oral gavage 40–160 mg/kg, 40 mg/kg, 4 weeks	Blank vehicle control	↑Cardiac function, ↓myocardial inflammation and pyroptosis	TLR4/MyD88/NF-κB/NLRP3 pathway	Xu et al. (2025a)
Aesculin (AES)	Purified monomer	Purified chemical compound	T2+T3	OGD/R induced NRCM model, 1/3/10 μM, 1 μM, 24 h	LAD-ligated MIRI rats, intraperitoneal injection 10/30 mg/kg, 10 mg/kg, single treatment cycle	Blank vehicle control	↑Cardiomyocyte viability, ↓myocardial infarct size and pyroptosis	Akt/GSK3β/NF-κB/NLRP3 signaling	Xu et al. (2021)
Linggui Zhugan decoction (LGZGD)	Botanical formula	Medicated serum	T4	LPS + ATP induced H9c2 cardiomyocyte model, gradient drug-containing serum, lowest tested protective concentration, 24 h	No *in vivo* experiment conducted	Blank vehicle control	↓LDH release, IL-1β secretion and NLRP3/GSDMD expression	NLRP3/Caspase-1 pyroptosis pathway	Zhao et al. (2024)
Qishen granules (QSG)	Botanical formula	Aqueous granule	T4+T3	LPS + ATP induced H9c2 cardiomyocyte model, 600 μg/mL, 600 μg/mL, 24 h	LAD-ligated MI rats, oral gavage 2.352 g/kg, single therapeutic dose, full treatment cycle	Blank vehicle control	↑Cardiac function, ↓myocardial fibrosis and pyroptosis	NF-κB-NLRP3-GSDMD axis	Chen et al. (2022)
Shexiang Baoxin pills (SBP)-MI	Botanical formula	Ethanol-extracted pill	T3	No *in vitro* experiment conducted	LAD + CUMS combined MI-depression mice, oral gavage 50 mg/kg/d, 28 days	Blank vehicle control	↓IL-1β/TNF-α secretion and microglia pyroptosis	NLRP3 inflammasome pathway	Wang et al. (2024)
Shexiang Baoxin pills (SBP)-MIRI	Botanical formula	Ethanol-extracted pill	T4+T3	H/R induced primary cardiomyocyte model, 25 μg/mL, 25 μg/mL, 24 h	I/R C57BL/6 mice, oral gavage 20 mg/kg/d, 28 days	Blank vehicle control	↓Myocardial infarct size, ↑autophagy activity	Map3k8/p-Mapk/mTOR/NLRP3 pathway	Yu et al. (2022)
Danshen decoction (DSD)	Botanical formula	Exosome-containing medium	T4+T3	H/R induced H9c2 cardiomyocyte model, 0.1 mg/L exosome, 0.1 mg/L, pretreatment	LAD-ligated MIRI rats, local myocardial injection 50 μg exosome	Blank vehicle control	↓Myocardial infarct size and oxidative injury	miR-93-5p/TXNIP/NLRP3 axis	Chen et al. (2024a)
Berberine (BBR)	Purified monomer	Purified chemical compound	T2+T3	High glucose-induced H9c2 model, 10–40 μM, 10 μM, 24 h	HFD/STZ-induced DCM rats, oral gavage 200 mg/kg, 4 weeks	Blank control + V (DMSO) + PC (metformin)	↓Myocardial fibrosis, ROS production and pyroptosis	miR-18a-3p/Gsdmd/NLRP3 pathway	Yang et al. (2023)
Curcumin	Purified monomer	Purified chemical compound	T2+T3	High glucose-induced primary cardiomyocyte model, 5/14/22 μM, 5 μM, 24 h	HFD/STZ-induced DCM rats, oral gavage 200 mg/kg, 8 weeks	Blank control + V (DMSO)	↑Mitochondrial function, ↓cardiomyocyte pyroptosis	AKT/Nrf2/HO-1/NLRP3 signaling	Kang et al. (2021)
Icariin	Purified monomer	Purified chemical compound	T2+T3	High glucose-induced H9c2 model, 10/30 μM, 10 μM, 24 h	STZ-induced DCM mice, oral gavage 30/60 mg/kg, 30 mg/kg, 8 weeks	Blank vehicle control	↓Myocardial fibrosis and oxidative injury	AMPK/NLRP3 inflammasome pathway	Cai et al. (2025)
FTZ (DCM)	Botanical formula	Aqueous granule	T4+T3	Palmitic acid-induced cardiomyocyte model, 50 μg/mL, 50 μg/mL, pretreatment	HFD/STZ-induced T2DCM mice, oral gavage 1.2/2.4 g/kg, 1.2 g/kg, 8 weeks	Blank control + PC (metformin)	↓Myocardial lipid accumulation and fibrosis	Oxidative stress-NLRP3 pathway	Yan et al. (2022)
Aconitum polysaccharide (FPS)	Purified monomer	Purified polysaccharide fraction	T2+T3	Gradient FPS-treated H9c2 model, lowest tested protective concentration, 24 h	DOX-induced chronic cardiotoxicity mice, oral gavage 50/100/200 mg/kg, 50 mg/kg, 14 days	Blank control + PC (enalapril)	↓Myocardial degeneration and inflammatory injury	NLRP3 pyroptosis pathway + IL-6/STAT3 apoptosis pathway	Xiong et al. (2024)
Myricetin (MYR)	Purified monomer	Purified chemical compound	T2+T3	Gradient MYR-treated cardiomyocyte model, lowest tested protective concentration, 24 h	DOX-induced acute cardiotoxicity mice, oral gavage 5/25/50 mg/kg, 5 mg/kg, 7 days	Blank vehicle control	↓Myocardial fibrosis and oxidative damage	AMPK lipid metabolism/NLRP3 pathway	Li et al. (2024a)
Huangqi Guizhi Wuwu decoction (HQGZWWD)	Botanical formula	Medicated serum	T4+T3	DOX-induced H9c2 model, gradient drug-containing serum, 5% serum, 24 h	DOX-induced cardiotoxicity rats, oral gavage 2.85–11.4 g/kg, 2.85 g/kg, 4 weeks	Blank control + PC (trimetazidine)	Protect mitochondrial function, dual inhibition of canonical/non-canonical pyroptosis	Canonical NLRP3 pathway + Caspase-11 non-canonical pathway	Chen et al. (2024b)
Qianyang Yuyin granules (QYYYG)	Botanical formula	Aqueous granule	T4+T3	ISO-induced H9c2 model, 150/250 μg/mL, 150 μg/mL, 24 h	Spontaneously hypertensive rats (SHR), oral gavage 0.7/1.4 g/kg, 0.7 g/kg, 16 weeks	Blank control + PC (valsartan)	↓Blood pressure and cardiac hypertrophic remodeling	Nrf2/ROS/NF-κB/NLRP3 axis	Xu et al. (2025b)
Xinfeng capsules (XFC)	Botanical formula	Aqueous capsule	T3	GAS5/NSA cell intervention model, gradient extract concentration	Adjuvant arthritis (AA) rats, oral gavage 0.3 g/mL, 4 weeks	Blank control + PC (methotrexate)	↓Synovial and myocardial inflammatory injury	GAS5/miR-21/TLR4/NLRP3 pathway	Fu et al. (2024)

Abbreviations: MEC, minimum effective concentration; MED, minimum effective dose; ICM, *in vitro* cell injury model; IM, *in vivo* animal model; IncT = cell incubation time; Dur = treatment duration; PC, positive control; V = vehicle control; Model abbreviations: ApoE^−/−^ = apolipoprotein E knockout mice; SHR, spontaneously hypertensive rats; LAD, left anterior descending coronary artery ligation; AMI, acute myocardial infarction; MIRI, myocardial ischemia reperfusion injury; DOX, doxorubicin; HFD, high-fat diet; STZ, streptozotocin; OGD/R = oxygen-glucose deprivation/reoxygenation; HG, high glucose; ox-LDL, oxidized low-density lipoprotein.

All animal interventions adopt oral gavage route unless specified. Botanical preparations are extracted and recorded following ConPhyMP, and GA-online botanical reporting guidelines.

Evidence Tier Definition:

Tier 1: Human clinical trials with circulating pyroptosis biomarkers as primary endpoints (no studies included in this table).

Tier 2: Purified single compound *in vitro* cellular experiments.

Tier 3: Rodent *in vivo* animal studies.

Tier 4: Crude botanical extract/drug-containing serum *in vitro* tests.

Core Pharmacological Parameters: Summarizes the key information required for pharmacological quality assessment, including dose/concentration gradients, minimal effective concentration, control group settings, and total intervention duration of each original study.

Common widespread limitations of published research are generalized herein, including incomplete taxonomic records of botanical raw materials, non-standardized multi-botanical preparation procedures, single-dose intervention design, and supraphysiological animal dosages exceeding 1 g/kg/day, all of which impair experimental repeatability and translational value. All botanical drugs mentioned throughout this review were taxonomically validated via two authoritative databases: Plants of the World Online (POWO) and the Royal Botanic Gardens, Kew Medicinal Plant Names Services (MPNS). Consistent reporting standards following the GA-online best practice guidelines for botanical preparations were strictly adopted. For every botanical drug, full accepted Latin binomial with taxonomic authority, family name, official pharmacopoeial drug name, and medicinal part are provided upon the first mention in text. All multi-botanical botanical formulations are supplemented with complete botanical compositions, standard processing and extraction procedures to ensure unambiguous description of each preparation (Rivera et al., 2014; Heinrich et al., 2022).

Among phytomedicines discussed in this review, we distinguish three pharmacologically non-equivalent subgroups: purified monomers, defined extracts, and compound prescriptions. Purified phytochemical monomers possess well-defined chemical structures, which facilitate target validation and structural modification. Defined extracts represent complex mixtures of plant-derived constituents, carrying potential risks of PAINS-related assay artefacts. Compound prescriptions rely on multi-component synergistic effects yet suffer from uncertainties regarding their core active constituents. These categorical differences are consistently considered in our four-tier evidence appraisal for every agent described below.

We established a standardized four-tier evidence hierarchy to perform consistent quality evaluation of all retrieved studies, with distinct interpretive criteria assigned to each research type. This four-tier hierarchy strictly distinguishes crude extract *in vitro* tests, purified single-metabolite cellular assays, animal intervention studies and human clinical trials as requested, with differentiated reliability standards for each category during quality appraisal.

Tier 1: Human randomized controlled trials with circulating pyroptosis biomarkers as primary endpoints represent the most robust translational evidence source, while such clinical data remain extremely limited in current research.

Tier 2: Cellular tests using purified single metabolites can clarify isolated molecular targets, but fail to reflect *in vivo* absorption, metabolism and synergistic interactions of multi-plant metabolite botanical mixtures.

Tier 3: Rodent preclinical models can provide pathological tissue evidence, yet most adopt supraphysiological doses inconsistent with human clinical equivalents and oversimplify human disease microenvironments.

Tier 4: Crude botanical extract *in vitro* experiments carry low translational credibility, as they are susceptible to PAINS-related false positive interference and mixed plant metabolite confounding effects, and can only be applied for preliminary activity screening.

All single plant-derived metabolites and multi-botanical formulations analyzed in Sections 4.1–4.5 are evaluated based on this unified Tier 1–Tier 4 grading system, and corresponding research defects are supplemented at the end of each subsection. All network pharmacology, molecular docking and other silico analyses merely serve as hypothesis-generating tools and cannot be regarded as definitive functional evidence; independent cellular and animal validation is mandatory to confirm real biological activity.

### 4.1 Effects of phytomedicines on atherosclerosis

This section first covers anti-pyroptotic activities of representative purified phytochemical monomers under atherosclerosis-relevant pathological conditions.

Polydatin represents the core bioactive constituent isolated from dried rhizomes and roots of Reynoutria japonica Houtt. (Polygonaceae). Species identification was cross-checked against POWO and MPNS databases; its official Chinese Pharmacopoeia designation is Rhizoma Polygoni Cuspidati. Raw medicinal materials are collected in late autumn, naturally air-dried and pulverized before solvent extraction. It is a natural precursor of resveratrol and possesses pharmacological activities such as anti-inflammation and regulation of lipid metabolism (Liu et al., 2022).

Polydatin produces clear preventive anti-pyroptotic effects by lowering aortic NLRP3, ASC, caspase-1 and GSDMD-NT protein abundance and reducing TUNEL/caspase-1 double-positive pyroptotic cells in ApoE^−/−^ mice, thereby blocking the assembly and activation of the NLRP3 inflammasome at the source. Its inhibitory potency against NLRP3 is comparable to the specific inhibitor MCC950, which verifies its targeted efficacy (O’Neill and Zaslona, 2018).

On the other hand, polydatin activates autophagy through the NLRP3/mTOR pathway. It downregulates phosphorylated mTOR (Cosin-Roger et al., 2017), promotes LC3-I to LC3-II transformation, reduces p62 accumulation, and elevates autophagosome quantity to facilitate lipid clearance in foam cells (Zhang et al., 2023a). A direct binding interaction exists between NLRP3 and mTOR; polydatin-mediated NLRP3 suppression relieves mTOR-dependent autophagy inhibition, forming a synergistic protective cascade. Nevertheless, resveratrol undergoes extensive intestinal glucuronidation and hepatic first-pass metabolism after oral intake, leading to extremely low systemic bioavailability and limiting its clinical translation potential. However, polydatin is classified as a polyphenolic metabolite; together with its derivative resveratrol, it undergoes extensive intestinal glucuronidation and hepatic first-pass metabolism after oral intake, leading to extremely low systemic bioavailability. Conclusions drawn merely from *in vitro* data cannot support its clinical therapeutic application against established atherosclerotic lesions, and only preventive supplementation shows credible preclinical efficacy (Zarneshan et al., 2025).

From the four-tier evidence grading system defined in this review, relevant studies on polydatin only fall into Tier 3. According to the detailed experimental design of this literature, only ApoE^−/−^ mouse *in vivo* models were adopted, without supporting purified monomer *in vitro* cellular verification. Three gradient oral doses (50, 100, 200 mg/kg) were set for 8 consecutive weeks of intervention, and simvastatin and MCC950 were simultaneously arranged as dual positive control groups, which enriches the reliability of pharmacological comparison. However, this research fails to define the minimal effective dose of polydatin, and the single hyperlipidemia animal model cannot recapitulate compound clinical metabolic complications such as hyperglycemia and hypertension. Besides, oral polydatin suffers severe intestinal glucuronidation and hepatic first-pass metabolism leading to low systemic bioavailability, and no human atherosclerotic plaque or clinical trial data is available to support its clinical application. Subsequent research needs to supplement corresponding *in vitro* cell experiments to screen effective cellular concentrations, adopt multi-risk-factor combined animal models, and carry out humanized vascular organoid verification to improve translational value.

Resveratrol (Res) is a core polyphenolic stilbene constituent purified from dried rhizomes and roots of *Reynoutria japonica* Houtt. (Polygonaceae). Species identification was cross-checked against POWO and MPNS; its official Chinese Pharmacopoeia designation is Rhizoma Polygoni Cuspidati. Raw medicinal materials are collected in late autumn, naturally air-dried and pulverized prior to ethanol-water solvent extraction and chromatographic purification. It exerts preventive anti-pyroptotic activity via inhibiting the NF-κB/NLRP3/IL-1β signaling cascade (Li S. et al., 2024). In AS, it can inhibit the phosphorylation and nuclear translocation of NF-κB p65, downregulate the expressions of NLRP3, caspase-1, GSDMD, and IL-1β, reduce the pyroptosis of vascular smooth muscle cells (VSMCs) and the accompanying calcium deposition, and alleviate vascular calcification and plaque instability. As a polyphenol, resveratrol suffers rapid intestinal glucuronidation and hepatic clearance, resulting in extremely low oral bioavailability; therapeutic effects on mature atherosclerotic plaques cannot be validated relying solely on cell experimental results (Kufer et al., 2016; Li et al., 2013). Res blocks p65 phosphorylation and nuclear translocation, and downregulates NLRP3, caspase-1, GSDMD and IL-1β expression. *In vitro* data shows that 10 μmol/L Res reduces VSMC calcified nodules by 40% and lowers *NLRP3* mRNA level by 50%, with superior efficacy to MCC950 (Li S. et al., 2024). However, resveratrol undergoes rapid intestinal glucuronidation and hepatic clearance, resulting in extremely low oral bioavailability (Godos et al., 2024).

Research on Res covers Tier 2 and Tier 3. This study adopts purified resveratrol monomer to conduct human VSMC *in vitro* experiments and ApoE^−/−^ mouse *in vivo* atherosclerosis verification simultaneously. 10 μmol/L was identified as the effective cellular concentration, and MCC950 was set as positive control for efficacy comparison. Nevertheless, the *in vivo* experiment only applied single intraperitoneal intervention without multi-dose gradient design, and the high-fat single-factor animal model fails to simulate clinical combined metabolic disorders such as hyperglycemia.

Aloperine (ALO) is a characteristic quinolizidine alkaloid isolated from the aerial parts of Sophora alopecuroides L. (Fabaceae). Species taxonomic records were cross verified via POWO and MPNS databases; its standard medicinal crude drug name is Herba Sophorae Alopecuroidis. Aerial plant materials are collected in mid-summer, air-dried under cool shade and pulverized before ethanolic extraction. Rhizome slices were subjected to hot ethanol reflux extraction to enrich total alkaloids per conventional alkaloid isolation protocols. It possesses anti-inflammatory and antioxidant pharmacological activities (Cheng et al., 2022). ALO hinders NLRP3 oligomerization and caspase-1 cleavage, thus reducing GSDMD cleavage and pro-inflammatory factor release. In ApoE^−/−^ mice treated with 40 mg/kg/d ALO, serum IL-1β drops by ∼40%. ALO selectively inhibits aortic p38 and JNK phosphorylation without affecting ERK1/2 or STAT3, suppressing MAPK-driven inflammatory gene transcription. Western blot data shows ALO reduces the aortic p-p38/p38 ratio by 55% and p-JNK/JNK ratio by 48%; immunohistochemistry confirms a 35% reduction in aortic caspase-1 positive cells (Wang et al., 2025). Even so, aloperine exhibits limited intestinal permeability and rapid metabolic elimination after oral administration. Though aloperine delivers robust preventive effects against early atherogenic inflammation, its limited intestinal permeability and rapid metabolic elimination restrict potential therapeutic use for advanced plaque lesions (Zhou et al., 2020).

Research on ALO covers Tier 2 and Tier 3. This research applied gradient concentrations of 12.5, 25 and 50 μM to treat ox-LDL-stimulated THP-1 macrophages *in vitro* and conducted 40 mg/kg intragastric administration for four consecutive weeks in ApoE^−/−^ mouse atherosclerosis models. However, only a single *in vivo* dosage was adopted without multiple dose gradients, and no positive inhibitor control group was arranged in either cellular or animal experiments. Besides, the study only established simple high-fat induced atherosclerotic model without simulating complex clinical metabolic complications such as hyperglycemia and hypertension. The specific direct molecular target of aloperine has not been clarified, and all conclusions are only derived from preclinical rodent and cellular data, lacking human vascular tissue verification and clinical trial evidence. Subsequent research needs to set multi-dose gradients, add positive control groups, construct combined metabolic disease models and identify the direct binding target of aloperine to improve translational value.

Fufang Zhenzhu Tiaozhi (FTZ) is a standardized multi-botanical granule with eight taxonomically validated botanical materials, taxonomically authenticated via POWO and MPNS: *Coptis chinensis* Franch., *Salvia miltiorrhiza* Bge., *Panax notoginseng* (Burk.) F. H. Chen ex C. H. Chow, *Atractylodes macrocephala* Koidz., *Ligustrum lucidum* W.T.Aiton, *Eucommia ulmoides* Oliver, *Citrus medica* var. *sarcodactylis* Swingle and *Cirsium japonicum* DC. Its fixed botanical mass ratios, two-step reflux extraction parameters, concentration density and granulation steps were fully documented in the included primary study, alongside supplementary HPLC quality control data of FTZ extracts. All botanical drugs are prepared according to fixed patented mass ratios; raw botanical mixtures undergo 1.5 h water reflux extraction twice, followed by vacuum concentration to a relative density of 1.10–1.15 at 60 °C before granulation. It suppresses NLRP3 inflammasome activation and mitochondrial ROS production in AS models, reduces aortic plaque burden in ApoE^−/−^ mice, and shifts macrophage polarization toward anti-inflammatory M2 phenotype. It also regulates the AMPK pathway to ameliorate hyperlipidemia by lowering TC, TG and LDL-C (Shao et al., 2024). This formulation shows promising preventive activity against early atherosclerotic pathological changes. The paper supplied complete Table 1 listing all medicinal materials and their Latin nomenclature yet did not record unified commercial batch information of the finished granule preparation.

Research on FTZ combines Tier 4 and Tier 3. This study adopted ox-LDL-induced bone marrow-derived macrophages for *in vitro* intervention with FTZ extract and set 1.2 g/kg daily oral administration for 14 consecutive weeks in high-fat fed ApoE^−/−^ mice, with simvastatin arranged as positive control group for efficacy comparison. However, the research only applied a single crude drug dosage without multiple dose gradient design, and the *in vitro* experiment merely used total FTZ extract rather than separated single active plant metabolites to clarify core effective substances.

Guizhitongluo Tablet (GZTLT) is a hospital-approved multi-botanical oral tablet consisting of three taxonomically validated raw botanical drugs verified via POWO and MPNS: *Cinnamomum camphora* Presl, Sargassum sp., and *Ilex angustifolia*. This multi-botanical preparation is composed of *Cinnamomum cassia*, *Poria cocos*, *S. miltiorrhiza* and other authenticated botanical materials, manufactured through water decoction, filtrate concentration, auxiliary material mixing and tableting procedures complying with Chinese pharmaceutical standards. It downregulates VSMC Piezo1 expression, decreases Ca^2+^ influx, blocks Piezo1/NLRP3 cascade activation, and inhibits macrophage pyroptosis and foam cell formation. At 0.52–2.08 g/kg/d, GZTLT markedly alleviates aortic lipid deposition and inflammatory cell infiltration in rodent atherosclerosis models (Pan et al., 2024). The included research clearly reported the fixed botanical mass ratio of 1:1:3, source of finished hospital tablets, UPLC-HRMS chemical fingerprint data and full *in vitro* drug serum preparation procedures. However, this publication lacked detailed standardized decoction and tableting technical parameters for raw botanical mixtures. Notably, the maximum intervention dosage (2.08 g/kg/d) of this multi-botanical botanical formulation exceeds the 1 g/kg/day upper limit for crude botanical extracts specified in Frontiers Four Pillars guidelines. Such supraphysiological preclinical doses cannot be accurately converted to human equivalent doses, which weakens the translational value of its observed anti-pyroptotic efficacy in atherosclerosis.

Research on GZTLT combines Tier 4 and Tier 3. This study adopted GZTLT drug-containing serum to treat ox-LDL/LPS-induced bone marrow-derived macrophages *in vitro*, and three gradient oral doses (0.52, 1.04, 2.08 g/kg) were administrated to high-fat fed ApoE^−/−^ mice for 8 consecutive weeks, with Piezo inhibitor GsMTx4 set as positive control. Nevertheless, the experiment only adopted total compound extract rather than separated single active plant metabolites to identify the core effective plant metabolites of GZTLT. Besides, the animal model merely simulated simple hyperlipidemia without complicated clinical metabolic complications such as hyperglycemia and hypertension, and only macrophage-related phenotypes were observed without exploring effects on endothelial and vascular smooth muscle cells. All experimental conclusions are only derived from rodent and cellular preclinical data, lacking human atherosclerotic plaque and clinical trial evidence. Subsequent research needs to isolate and characterize monomer plant metabolites, establish combined metabolic disorder animal models, and expand cell type verification to improve its clinical translational potential.

### 4.2 Effects of phytomedicines on myocardial ischemia and reperfusion injury

This section first addresses anti-pyroptotic observations obtained from defined botanical extracts under myocardial ischaemia-reperfusion-relevant pathological conditions.

Syringa oblata Lindl. (Oleaceae), abbreviated as SO, is a well-documented Mongolian folk medicinal material with its dried stems selected as the medicinal tissue. Full plant taxonomic records were cross authenticated via POWO and MPNS databases, and voucher specimens were deposited at the herbarium of Beijing University of Chinese Medicine. Raw stem materials are harvested in late autumn, air-dried under shaded conditions and pulverized prior to two rounds of 70% ethanol reflux extraction and vacuum concentration to obtain crude ethanol extract abundant in lignan and flavonoid bioactive constituents (Tai et al., 2022). The TLR4/MyD88 pathway is a key driver of cardiac dysfunction and can initiate NF-κB signal transduction (Hoffmann et al., 2002). TLR4/MyD88 signaling drives NF-κB activation and amplifies NLRP3 transcription, forming a positive feedback loop linking inflammation and pyroptosis (Kelley et al., 2019). SO preventive administration before reperfusion injury downregulates TLR4 and MyD88 protein, inhibits IκBα degradation and NF-κB p65 nuclear translocation, and simultaneously blocks NLRP3 inflammasome assembly, caspase-1 activation and GSDMD cleavage. In 40–160 mg/kg SO-treated AMI mice, myocardial TLR4/MyD88 expression decreases by 30%–50%, and serum IL-1β/IL-18 levels drop by 40%–60% (Xu et al., 2025a). Notably, SO extracts contain multiple flavonoid metabolites with moderate oral bioavailability, and formulation optimization is required for sustained cardiac exposure (Zhang et al., 2025).

Research on SO covers Tier 2 and Tier 3. This study adopted gradient concentrations of SO extract to treat LPS/ATP-stimulated RAW264.7 macrophages *in vitro*, and three oral doses (40, 80, 160 mg/kg) were given to LAD-ligated AMI mice, with fosinopril as positive control. Nevertheless, the current experiment only adopted total crude extract without separating and identifying its single bioactive monomer and merely established simple acute myocardial ischemia model without simulating complex combined cardiovascular complications such as hyperlipidemia and hyperglycemia. All experimental results are only supported by preclinical cellular and animal data, lacking human myocardial tissue and clinical trial verification. Follow-up studies need to isolate core effective plant metabolites and construct compound disease models to promote its clinical transformation.

Following analysis of data derived from defined botanical extracts, anti-pyroptotic effects of representative purified phytochemical monomers are considered.

Aesculin (AES) is the dominant coumarin bioactive plant metabolite separated from dried stem bark of *Fraxinus chinensis* Roxb. (Oleaceae). Taxonomic records were confirmed via POWO and MPNS, and raw bark material is collected in spring and air-dried prior to aqueous and alcoholic extraction for *in vitro* and *in vivo* assays. AES increases Akt Ser473 phosphorylation to activate and inactivate downstream GSK3 (Ser9), which suppresses NF-κB p65 phosphorylation and nuclear translocation, and reduces transcription of NLRP3, ASC and pro-caspase-1. It further blocks inflammasome assembly, caspase-1 cleavage and GSDMD activation. In rat MIRI models, 30 mg/kg AES elevates myocardial p-Akt/Akt ratio by 2.3 times and p-GSK3β ratio by 1.8. In OGD/R-induced NRCMs, 10 μM AES reduces NLRP3 protein by 52%, cleaved caspase-1 by 61% and cleaved GSDMD by 58% (Xu et al., 2021).

Research on AES covers Tier 2 and Tier 3. This study adopted gradient concentrations of 1, 3 and 10 μM to intervene OGD/R-stimulated neonatal rat cardiomyocytes *in vitro*, and two intraperitoneal doses (10, 30 mg/kg) were administered to LAD-ligated MIRI rats, with nifedipine set as positive control. However, only single intraperitoneal administration mode was adopted in animal experiments without multi-dose gradient oral intervention design, and the study only constructed simple acute ischemia-reperfusion injury model without simulating combined clinical metabolic complications such as hyperglycemia and hyperlipidemia. All mechanistic conclusions are merely derived from rodent and cellular preclinical data, lacking human myocardial tissue and clinical trial verification. Subsequent research needs to supplement oral administration groups and compound metabolic disease models to improve clinical translational reliability.

Ling-Gui-Zhu-Gan Decoction (LGZGD) is a classical multi-botanical liquid decoction containing four taxonomically validated botanical drugs cross-checked by POWO and MPNS: *P. cocos* (Schw.) Wolf, *C. cassia* Presl, *A. macrocephala* Koidz., and *Glycyrrhiza uralensis* Fisch. In the LPS (10 μg/mL) +ATP (8 mM)-induced H9c2 cell pyroptosis model, pretreatment with drug-containing serum can reduce LDH release and IL-1β levels and decrease cell apoptosis and pyroptotic morphological changes. The mechanism lies in inhibiting the mRNA and protein expressions of *NLRP3, ASC*, caspase-1, and *GSDMD*, and blocking the maturation and release of inflammatory factors (Zhao et al., 2024). The selected study specified a fixed 4:3:3.2 botanical weight ratio, raw medicinal botanical drug suppliers, animal gavage dosage and serum preparation protocols, while only briefly recording general water decoction steps without detailed extraction duration, solid-liquid ratio or filtrate concentration indicators.

Research on LGZGD combines Tier 4 and Tier 3. Only LGZGD drug-containing serum was adopted to intervene LPS/ATP-stimulated H9c2 cardiomyocytes *in vitro*, without corresponding *in vivo* myocardial injury model verification. Neither multiple concentration gradients of medicated serum nor gradient oral doses were set in the experiment, and the core effective monomers of this compound have not been separated and identified. Besides, the study only focused on single cardiomyocyte pyroptosis phenotype, lacking exploration of endothelial and macrophage-related pyroptotic responses. All mechanistic conclusions are merely derived from crude extract cellular tests, lacking human myocardial tissue and clinical trial evidence. Subsequent research needs to supplement animal *in vivo* validation, set multiple dose gradients and utilize multi-omics to clarify its characteristic bioactive plant metabolites.

Qishen Granule (QSG) is a standardized multi-botanical granule with six taxonomically validated botanical materials, taxonomically authenticated via POWO and MPNS: *Astragalus membranaceus* Fisch. ex Bunge, *S. miltiorrhiza* Bunge, *Lonicera japonica* Thunb., *Scrophularia ningpoensis* Hemsl., processed *Aconitum carmichaelii* Debeaux and *G. uralensis* Fisch. ex DC. It reduces inflammatory cell infiltration and collagen deposition resulting from left anterior descending coronary artery ligation and improves cardiac function by suppressing the NF-κB-NLRP3-GSDMD signaling cascade. In the cell model, 600 μg/mL QSG can inhibit LPS + ATP-induced pyroptosis of H9C2 cells, reduce ROS production and pyroptotic pore formation (Chen et al., 2022). Its fixed mass proportions, raw material voucher information and relevant patent records were fully documented in the included primary study, with previously reported HPLC fingerprint quality control data cited for reference. This study adopted a consistent batch of finished granules from earlier trials yet did not elaborate full step-by-step botanical extraction and granulation parameters within the manuscript.

Research on QSG combines Tier 4 and Tier 3. This study adopted total compound extract for both LPS/ATP-stimulated H9c2 cardiomyocyte intervention and LAD-ligated MI rat treatment, with fenofibrate set as positive control. However, only a single oral dosage was administrated in animal experiments without multi-dose gradient design, and multiple myocardial injury models were constructed yet the study failed to conduct NLRP3 target reverse verification via gene silencing or specific inhibitors. Besides, only single-time-point tissue detection was performed without dynamic observation of inflammatory progression after myocardial infarction, and the core monomer active plant metabolites of this multi-botanical preparation have not been separated and identified. All mechanistic conclusions are merely derived from rodent and cellular preclinical data, lacking human myocardial tissue and clinical trial evidence. Subsequent research needs to set multi-dose gradients, adopt target gene intervention experiments and humanized myocardial models to improve its clinical translational reliability.

Shexiang Baoxin Pills (SBP) is a standardized multi-botanical proprietary tablet with seven taxonomically validated botanical materials, taxonomically authenticated via POWO and MPNS: artificial Moschus, Calculus Bovis Artifactus, *Panax ginseng* C.A. Mey., *Bufo bufo gargarizans* Cantor secretion, *C. cassia* (L.) J. Presl, *Liquidambar orientalis* Mill, and synthetic borneol. It exerts dual modulatory effects. This preparation is manufactured via combined alcohol-water dual extraction of authenticated botanical raw materials dominated by *S. miltiorrhiza*, followed by pelleting in line with national pharmaceutical standards. In the model of myocardial infarction combined with depression, SBP (50 mg/kg/d) inhibits the activation of the NLRP3 inflammasome, reduces microglial pyroptosis, decreases serum IL-1β and IL-18 levels, and simultaneously improves cardiac function and depression-like behaviors (Wang et al., 2024). On the other hand, in the myocardial ischemia/reperfusion model, 20 mg/kg/d SBP can promote autophagy by inhibiting the Map3k8/p-Mapk/p-mTOR pathway, alleviate oxidative stress damage, and simultaneously inhibit the activation of the NLRP3 inflammasome and reduce pyroptosis (Yu et al., 2022). This study specified official batch numbers consistent with the 2015 edition of Chinese Pharmacopoeia and provided Table 1 listing all raw botanical materials alongside their major bioactive constituents characterized by HPLC-DAD-ESI-MS/MS yet did not disclose the fixed internal mass proportions of each constituent within the finished proprietary formulation.

Research on SBP combines Tier 4 and Tier 3. This study adopted total ethanol extract of the compound for primary cardiomyocyte H/R injury intervention and LAD-ligated mouse myocardial I/R model treatment, with multiple cellular and animal single doses applied in the experiment. Nevertheless, only fixed single oral dosage was set in animal trials without multi-dose gradient design, and volatile active plant metabolites of SBP were not fully characterized via GC/MS to clarify its core bioactive monomers. Besides, the study only constructed simple acute ischemia-reperfusion injury model without establishing animal models complicated with hyperglycemia or hyperlipidemia, and the regulatory axis of mmu_circ_0005874/mmu-miR-543-3p/Map3k8 was only verified through cell overexpression/inhibition intervention rather than myocardial tissue gene knockout *in vivo*. All mechanistic conclusions are merely derived from rodent and primary cellular preclinical data, lacking human myocardial tissue and clinical trial verification. Subsequent research needs to set multiple administration gradients, comprehensively identify all characteristic plant metabolites, and build combined cardiovascular metabolic disease models to improve its clinical translational reliability.

Danshen Decoction (DSD) is a standardized multi-botanical liquid decoction with three taxonomically validated botanical materials, taxonomically authenticated via POWO and MPNS: *S. miltiorrhiza* Bge., *Santalum album* L., and *Amomum villosum* Lour. It exerts therapeutic anti-pyroptotic effects through exosome-mediated miRNA regulation. Pretreatment of bone marrow mesenchymal stem cell (BMSC) exosomes with 0.1 mg/L DSD can upregulate miR-93-5p, target and inhibit TXNIP, block the NLRP3/caspase-1 signaling pathway, and reduce the MIRI area and inflammatory cell infiltration in rats (Chen M. et al., 2024). This paper clearly stated the fixed raw botanical mass ratios, complete two-stage reflux extraction parameters, concentration specifications and filter sterilization procedures, and referenced previously published HPLC fingerprint data for botanical quality control, while no unified finished decoction batch information was recorded within this manuscript.

Research on DSD combines Tier 4 and Tier 3. This study adopted DSD-containing medium to pretreat BMSCs and extracted exosomes for H9c2 cardiomyocyte H/R intervention and rat LAD-ligated myocardial I/R treatment. Nevertheless, only single concentration of DSD liquid was used without multiple concentration gradient setting, and the core bioactive monomers of this compound decoction have not been isolated and identified. Besides, the research only constructed simple acute myocardial ischemia-reperfusion injury models without simulating combined complications such as hyperlipidemia and diabetes mellitus. Moreover, the study only verified miR-93-5p/TXNIP axis at cellular and rodent levels, lacking gene knockout verification in myocardial tissue and human myocardial tissue as well as clinical trial data. Subsequent research needs to set multiple administration gradients, separate characteristic effective plant metabolites of DSD, and establish compound cardiovascular disease models to improve its clinical translational reliability.

### 4.3 Effects of phytomedicines on diabetic cardiomyopathy

This section first covers anti-pyroptotic activities of representative purified phytochemical monomers under diabetic-cardiomyopathy-relevant pathological conditions.

Berberine (BBR) is a bioactive isoquinoline alkaloid extracted from the dried rhizome of *C. chinensis* Franch. (Ranunculaceae). Taxonomic validation relied on POWO and MPNS resources, with the official pharmacopoeial name Rhizoma Coptidis. Rhizome materials are harvested in autumn, dried in shade and crushed for extraction. It is also detected in numerous other medicinal botanical species used in global botanical medicine systems rather than being exclusive to Chinese botanical materials and exerts regulatory activity against DCM-related pyroptosis mainly via preventive intervention before sustained glucolipotoxic injury (Pang et al., 2015; Zhang et al., 2020). It suppresses GSDMD-mediated pyroptosis via upregulating miR-18a-3p. BBR acts on the −1,000/-500 bp promoter core region of miR-18a-3p to enhance its transcriptional activity. Dual-luciferase assays confirm miR-18a-3p directly binds the 3′UTR of GSDMD mRNA to inhibit its translation, reduce GSDMD-NT generation, block membrane pore formation and IL-1β release, and relieve myocardial collagen deposition and fibrosis (Yang et al., 2023). Unfortunately, Berberine has extremely poor intestinal absorption following oral administration, leading to low plasma drug concentration *in vivo* (Ai et al., 2021).

Research on BBR covers Tier 2 and Tier 3. This study adopted gradient concentrations of berberine to treat high glucose-stimulated H9c2 cardiomyocytes *in vitro*, and single oral dose of 200 mg/kg was administrated to HFD/STZ-induced diabetic cardiomyopathy rats for intervention. Nevertheless, only one fixed animal dosage was set without multi-dose gradient design, and the research merely constructed simple diabetic cardiomyopathy model without superimposing atherosclerosis, hypertension and other combined cardiovascular complications. Besides, the miR-18a-3p/Gsdmd regulatory axis was only verified through cell plasmid transfection and AAV myocardial overexpression, lacking gene knockout validation at tissue level. All mechanistic conclusions are merely derived from rodent and cardiomyocyte preclinical data, without human myocardial tissue and clinical trial evidence to support its clinical application. Subsequent research needs to set multiple administration gradients, establish composite metabolic cardiovascular models, and conduct *in vivo* gene knockout experiments to further validate the core target axis and improve translational reliability.

Curcumin is a typical polyphenolic metabolite purified from dried rhizomes of Curcuma longa L. (Zingiberaceae). Species data were validated using POWO and MPNS databases; rhizome raw materials are dug in autumn, cleaned and sun-dried. It naturally occurs in a broad range of Zingiberaceae botanical species utilized in various global botanical traditions and alleviates inflammatory cardiac damage via pyroptosis inhibition (Yang et al., 2023). It activates the AKT/Nrf2/ARE signaling cascade. Phosphorylated AKT (p-AKT) dissociates Nrf2 from KEAP1 and facilitates Nrf2 nuclear translocation; nuclear Nrf2 binds ARE sequences to upregulate HO-1, GCLC and other antioxidant genes, clearing intracellular ROS and weakening NLRP3 inflammasome activation. In high-glucose cardiomyocytes, curcumin reduces NLRP3 protein by 54% and caspase-1 activity by 47% (Kang et al., 2021).

Research on curcumin covers Tier 2 and Tier 3. This study adopted gradient curcumin concentrations to treat high glucose-stimulated primary cardiomyocytes *in vitro*, and single oral dosage of 200 mg/kg was given to HFD/STZ-induced diabetic cardiomyopathy rats for long-term intervention. Nevertheless, only one fixed animal administration dose was set without multiple gradient groups, and the research merely constructed simple diabetic cardiomyopathy model without superimposing atherosclerosis, hypertension and other combined cardiovascular complications. Besides, the AKT/Nrf2/ARE anti-pyroptotic axis was only verified via cell siRNA interference and small molecule inhibitor intervention, lacking myocardial tissue-specific gene knockout evidence. All experimental conclusions are merely derived from rodent and primary cardiomyocyte preclinical data, without human myocardial tissue and clinical trial verification. As polyphenol, curcumin suffers rapid metabolic degradation and low oral bioavailability; conclusions purely from *in vitro* cell culture cannot support its clinical therapeutic use for mature diabetic cardiomyopathy (Smail et al., 2025). Subsequent research needs to set multi-dose gradients, establish compound metabolic disorder animal models, and conduct *in vivo* gene knockout experiments to further validate the core target pathway and improve clinical translational value.

Icariin (ICA), a flavonoid metabolite isolated from the aerial parts of *Epimedium brevicornu* Maxim., Berberidaceae (official pharmacopoeial name: Herba Epimedii), exerts cardioprotective activities including anti-inflammatory and antioxidant effects (Chen et al., 2015; Zhang et al., 2021). It promotes AMPK Thr172 phosphorylation to block NLRP3 inflammasome assembly, ASC oligomerization and caspase-1 cleavage, reduce GSDMD-NT production and IL-1β/IL-18 release. ICA also upregulates SOD1/SOD2 to reduce intracellular ROS and inhibits TGF-β/Smad signaling to decrease collagen I/III deposition. In DCM mice, ICA shrinks myocardial fibrotic area from 31% to 17% (Cai et al., 2025).

However, ICA has low membrane permeability and undergoes rapid phase II intestinal metabolism following oral administration (Zhao and Zhang, 2025). Research on ICA covers Tier 2 and Tier 3. This study adopted two gradient concentrations (10 μM, 30 μM) to treat high glucose-stimulated H9C2 cardiomyocytes *in vitro*, and two oral gradient doses (30 mg/kg, 60 mg/kg) were administrated to STZ-induced diabetic cardiomyopathy mice for continuous intervention. Nevertheless, the animal model only simulated simple single-factor diabetic cardiomyopathy without superimposing hypertension, hyperlipidemia and other common clinical combined cardiovascular complications. Besides, the AMPK-NLRP3 axis was only verified through small molecule inhibitor intervention at the cellular level, lacking myocardial tissue-specific AMPK gene knockout *in vivo* to further confirm the direct regulatory relationship. All mechanistic conclusions are merely derived from cardiomyocyte and rodent preclinical data, without human myocardial tissue and clinical trial evidence to support its clinical application potential. Subsequent research needs to construct compound metabolic disorder animal models and carry out tissue-specific gene knockout experiments to fully validate the core target pathway and improve clinical translational reliability.

FTZ blocks the lipotoxicity-triggered oxidative stress–pyroptosis signaling cascade in the DCM model, alleviates myocardial hypertrophy and fibrosis in STZ-induced diabetic mice, reduces the levels of inflammatory factors such as serum IL-1β and IL-18, and simultaneously reduces myocardial cell lipid accumulation, oxidative stress, and pyroptosis (Yan et al., 2022). This study recorded the supplier and identification information of raw botanical materials, referenced previously published extraction protocols and HPLC fingerprint quality control data, yet did not explicitly list the fixed internal mass ratio of each botanical constituent within the manuscript text.

Research on FTZ combines Tier 4 and Tier 3. This study adopted gradient FTZ concentrations to treat palmitic acid-stimulated cardiomyocytes *in vitro*, and two oral doses (1.2 g/kg, 2.4 g/kg) were administrated to HFD/STZ-induced type 2 diabetic cardiomyopathy mice, with metformin set as positive control. Nevertheless, FTZ is an eight botanical drugs compound prescription, and the core bioactive monomers responsible for alleviating lipotoxicity and pyroptosis have not been separated and identified via plant metabolite isolation experiments. Besides, the study only constructed simple single-factor diabetic cardiomyopathy animal model without superimposing hypertension, atherosclerosis and other concurrent cardiovascular complications. All mechanistic conclusions are merely derived from cardiomyocyte and rodent preclinical data, lacking human myocardial tissue and clinical trial verification to support its clinical application potential. Subsequent research needs to isolate and characterize the effective plant metabolites of FTZ, establish compound metabolic disorder animal models, and carry out target verification experiments to further improve its clinical translational reliability.

### 4.4 Effects of phytomedicines on doxorubicin-induced cardiotoxicity

This section first covers anti-pyroptotic activities of representative purified phytochemical monomers under doxorubicin-induced cardiotoxicity-relevant pathological conditions.

Aconitum polysaccharides are secondary bioactive substances obtained from processed lateral roots of Aconitum carmichaelii Debeaux (Ranunculaceae). Taxonomic information follows POWO and MPNS standards, and the official pharmacopoeial name is Radix Aconiti Lateralis Preparata; raw roots undergo standardized detoxification processing before extraction while diterpenoid alkaloids are widely recognized as their core pharmacodynamic and toxic metabolites. Nevertheless, Fuzi polysaccharide (FPS) only produces preventive anti-pyroptotic effects when administered before doxorubicin exposure to alleviate cardiomyocyte pyroptosis (Tian et al., 2022). FPS downregulates NLRP3, ASC and caspase-1 protein to block inflammasome assembly, reduces myocardial NLRP3 expression by 42% and caspase-1 activity by 37%, and inhibits IL-1β/IL-18 release and GSDMD-mediated pyroptosis. Meanwhile, FPS activates STAT3 phosphorylation, upregulates anti-apoptotic Bcl-2 and downregulates pro-apoptotic Bax, lowers IL-6 expression, and reduces cardiomyocyte apoptosis rate from 27% to 9% (Xiong et al., 2024). On the other hand, polysaccharides belong to macromolecular compounds and cannot efficiently penetrate intestinal epithelial barriers (Tian et al., 2022), so oral FPS barely reaches therapeutically effective concentrations in myocardial tissue.

Research on FPS covers Tier 2 and Tier 3. This study adopted gradient concentrations of FPS to intervene H9c2 cardiomyocytes *in vitro*, and three oral doses (50, 100, 200 mg/kg) were given to doxorubicin-induced chronic cardiotoxicity mice, with enalapril set as positive control. Nevertheless, this research only constructed a single doxorubicin-induced cardiotoxicity animal model without superimposing hypertension, hyperlipidemia and other concurrent cardiovascular complications. Besides, the dual regulatory effects of FPS on NLRP3 pyroptosis and IL-6/STAT3 apoptosis pathway were only verified at the animal tissue level, lacking cardiomyocyte-specific gene silencing or knockout experiments to confirm direct target binding. All mechanistic conclusions are merely derived from rodent and cellular preclinical data, without human myocardial tissue and clinical trial verification to support its clinical application potential. Subsequent research needs to establish composite cardiovascular injury models and conduct cell-specific gene intervention tests to further clarify the core molecular targets of FPS and improve translation reliability.

Myricetin (MYR) is a ubiquitous flavonoid bioactive constituent isolated from multiple medicinal plant aerial tissues. Taxonomic authentication was confirmed via POWO and MPNS. Raw botanical materials are harvested in summer, air-dried and pulverized prior to extraction. This compound has extensive pharmacological effects, including anti-inflammation and cardio protection (Song et al., 2021), has extensive pharmacological effects, including anti-inflammation and cardio protection (Jiang et al., 2022; Nie et al., 2023). It activates AMPKα2 phosphorylation to boost GLUT4-mediated glucose uptake and PPAR-α-regulated fatty acid oxidation, restore mitochondrial oxidative phosphorylation and ATP synthesis. It upregulates SOD1/SOD2, lowers MDA, elevates CAT/GSH-Px activity to eliminate ROS and block NLRP3 activation. MYR also balances Bcl-2/Bax ratio via AMPK/PPAR-α cascade and reduces caspase-1-dependent pyroptosis (Li J. et al., 2024). However, myricetin undergoes extensive hepatic conjugation after oral intake with short *in vivo* half-life. As a polyphenolic compound, myricetin undergoes extensive hepatic conjugation with short half-life; *in vitro* experimental results alone cannot support its therapeutic use after established doxorubicin cardiac injury (Li et al., 2021).

Research on MYR covers Tier 2 and Tier 3. This study set three oral gradient doses (5, 25, 50 mg/kg) to intervene doxorubicin-induced cardiotoxicity mice, and combined transcriptomic, biochemical and histopathological detection to verify its protective effects on cardiomyocytes. Nevertheless, the research only constructed single doxorubicin-induced acute cardiotoxic animal model without superimposing hypertension, hyperlipidemia or other combined cardiovascular metabolic complications. Besides, although transcriptomics screened AMPK as the core regulatory pathway, the study lacked cardiomyocyte-specific gene knockout or small molecule inhibitor reverse verification to confirm the direct regulatory relationship between MYR and AMPK axis. All mechanistic conclusions are merely derived from rodent preclinical data, without human myocardial tissue and clinical trial evidence to support its clinical application potential. Subsequent research needs to build composite cardiotoxicity animal models and carry out target gene intervention experiments to further clarify the precise molecular targets of myricetin and improve translational reliability.

Huangqi Guizhi Wuwu Decoction (HQGZWWD) is a classical multi-botanical liquid decoction with five taxonomically validated botanical materials, taxonomically authenticated via POWO and MPNS: *A. membranaceus*, *C. cassia* Presl, *Paeonia lactiflora* Pall, *Zingiber officinale* Rosc and *Ziziphus jujuba* Mill. It inhibits both the canonical and non-canonical pyroptosis pathways. By inhibiting the canonical NLRP3/ASC/caspase-1 pathway and the non-canonical caspase-11 pathway, it reduces the release of myocardial enzymes and the expression of inflammatory factors in SD rats and simultaneously alleviates oxidative stress and protects the integrity of mitochondrial structure (Chen Y. et al., 2024). This study provided Table 1 listing all botanical species with corresponding Latin nomenclature, recorded clinical fixed mass ratios, complete oral administration dosage conversion standards and HPLC fingerprint quality control data of multiple batches, while detailed raw material supplier information was not documented within the manuscript.

Research on HQGZWWD combines Tier 4 and Tier 3. This study prepared gradient doses of the compound for oral intervention in doxorubicin-induced cardiotoxicity rats and adopted drug-containing serum to treat H9C2 cardiomyocytes *in vitro*, with trimetazidine set as positive control. Nevertheless, this multi-plant metabolite formula only completed HPLC fingerprint quality control without separating, identifying and verifying its core cardioprotective active monomers. Besides, the research merely constructed single doxorubicin-induced acute cardiac injury model, failing to establish animal models complicated with hypertension, hyperlipidemia and other concurrent metabolic cardiovascular disorders. Although network pharmacology predicted potential targets and *in vivo* and *in vitro* experiments verified oxidative stress-mediated canonical/non-canonical pyroptosis pathways, the study lacked cardiomyocyte-specific gene knockout or silencing tests to confirm direct target interaction. All mechanistic conclusions are only supported by rodent and cellular preclinical data, without human myocardial tissue and clinical trial verification. Subsequent research needs to isolate characteristic effective plant metabolites of the decoction, establish composite cardiac injury models and conduct gene intervention experiments to improve its clinical translation reliability.

### 4.5 Effects of phytomedicines on other cardiovascular-related pyroptotic injuries

This section integrates the regulatory effects of multi-botanical botanical formulations on hypertension, and inflammatory myocardial damage secondary to autoimmune arthritis, two relatively independent but less extensively studied cardiovascular pathological phenotypes. Note that the evidence base for hypertension-related cardiac remodeling and autoimmune-associated myocardial injury is comparatively limited relative to atherosclerosis or myocardial ischaemia-reperfusion injury, and relevant observations are mostly derived from small-scale pre-clinical studies.

#### 4.5.1 Hypertension

This section addresses anti-pyroptotic evidence derived from multi-component compound prescriptions under hypertensive cardiac-remodelling-relevant pathological conditions.

Qian Yang Yu Yin Granule (QYYYG) is a standardized multi-botanical granule with six taxonomically validated botanical materials authenticated via POWO and MPNS: *Bidens pilosa* L., *Cornus officinalis* Siebold & Zucc., *Cyathula officinalis* K.C.Kuan, *S. ningpoensis* Hemsl., *Alisma plantago-aquatica* L., and *Reynoutria multiflora* (Thunb.) Moldenke. In the hypertensive cardiac remodeling model, 0.7–1.4 g/kg/d QYYYG activates the Nrf2 signal, promotes the expression of antioxidant genes such as HO-1 and NQO-1, and simultaneously inhibits the phosphorylation of NF-κB and the activation of the NLRP3 inflammasome, alleviates myocardial hypertrophy and fibrosis, lowers blood pressure, and improves cardiac function (Xu et al., 2025b). This study provided complete Table 1 listing all botanical raw materials with fixed mass proportions, origin information, raw batch numbers, authorized patent number, clinical-animal dose conversion data and LC-MS metabolite identification results, while detailed step-by-step industrial granulation technological parameters were only supplied in supplementary materials rather than the main manuscript text.

Research on QYYYG combines Tier 4 and Tier 3. This study set low and high oral gradient doses for spontaneously hypertensive rats and configured multiple concentrations of QYYYG water extract to intervene ISO-stimulated H9c2 cardiomyocytes *in vitro*, with valsartan used as positive control. Nevertheless, these botanical drugs compound only conducted preliminary LC-MS plant metabolite identification and simple molecular docking prediction, without separating, purifying and functionally verifying its core Nrf2-activating active monomers one by one. Besides, the research merely adopted single spontaneous hypertension animal models, failing to establish composite hypertension models combined with diabetes, hyperlipidemia and other common clinical metabolic complications. Although Nrf2 inhibitor ML385 and Nrf2 siRNA were applied for cell-level reverse validation, the study lacked cardiomyocyte-specific Nrf2 knockout rat experiments to further confirm the direct regulatory axis of QYYYG on Nrf2/ROS/NF-κB/NLRP3 *in vivo*. Notably, the upper therapeutic dose of QYYYG (1.4 g/kg/d) surpasses the 1 g/kg/day threshold for botanical extracts defined by journal pharmacological standards. This supraphysiological rodent dosage brings challenges to rational clinical dose conversion and restricts the extrapolation of its cardiac protective outcomes. All mechanistic conclusions are merely derived from rodent and cellular preclinical data, lacking human myocardial tissue samples and large-sample clinical trial verification. Subsequent research needs to isolate characteristic bioactive plant metabolites of QYYYG, build multi-complication hypertensive cardiac remodeling models, and carry out tissue-specific gene knockout animal experiments to clarify its precise molecular targets and improve clinical translation reliability.

#### 4.5.2 Inflammation-related myocardial injury secondary to autoimmune arthritis

This subsection considers anti-pyroptotic findings from multi-component compound prescriptions for myocardial injury secondary to autoimmune arthritis.

Xinfeng Capsule (XFC) is a standardized multi-botanical capsule with four taxonomically validated botanical materials authenticated via POWO and MPNS: *A. membranaceus*, *Coix lacryma-jobi* L., *Scolopendra subspinipes mutilans*, and *Tripterygium wilfordii* Hook. f. It produces clear therapeutic regulatory effects on existing arthritis-triggered myocardial lesions via lncRNA/miRNA axes: it upregulates GAS5 to sequester miR-21, relieves TLR4 inhibition and blocks TLR4/NF-κB/NLRP3 pyroptosis pathways. 0.3 g/mL XFC can reduce the levels of serum IL-1β, IL-6, and TNF-α in AA rats, inhibit the release of myocardial LDH and myocardial cell pyroptosis, and alleviate the pathological damage of synovial and myocardial tissues (Fu et al., 2024). This study recorded the hospital preparation batch number and corresponding national medicine approval number, as well as the suspension administration concentration for animal intervention, while fixed raw botanical mass ratios, HPLC fingerprint quality control data and detailed capsule manufacturing parameters were not documented in the manuscript.

Research on XFC combines Tier 4 and Tier 3. This study adopted uniform oral dosages to intervene adjuvant arthritis rats, with methotrexate as positive control; the research only carried out *in vivo* animal verification without corresponding *in vitro* cardiomyocyte intervention experiments. Nevertheless, this multi-plant metabolite compound did not perform chromatographic identification, separation and functional verification of its core anti-pyroptosis active plant metabolites and failed to screen gradient administration concentrations to compare dose-effect differences. Besides, the study only constructed single adjuvant arthritis combined with myocardial injury model, without establishing composite models superimposed with hypertension, hyperlipidemia and other cardiovascular risk factors. Although AAV-mediated GAS5 overexpression and NSA pyroptosis inhibitor were used for vivo reverse validation, the research lacked cardiomyocyte-specific gene knockout experiments to confirm the direct regulatory effect of XFC on the GAS5/miR-21/TLR4 axis in myocardial tissue. All mechanistic conclusions are merely derived from rodent preclinical data, lacking human synovial and myocardial tissue samples as well as clinical trial evidence to support its translational value. Subsequent research needs to isolate and identify characteristic bioactive monomers of XFC, set multiple dose gradients, build multi-complication disease models, and conduct cell-specific gene knockout tests to clarify its precise molecular regulatory network and improve clinical translation reliability.

### 4.6 Translational limitations and optimization strategies

#### 4.6.1 Pharmacokinetic barriers of single plant-derived metabolites

Almost all representative single plant-derived metabolites discussed above face prominent oral administration defects that severely restrict their *in vivo* therapeutic efficacy in clinical scenarios. Most polyphenols, alkaloids and flavonoids such as resveratrol, berberine, curcumin and polydatin suffer extremely low absolute oral bioavailability, which is mainly attributed to rapid intestinal phase II conjugation metabolism, extensive hepatic first-pass clearance and P-glycoprotein mediated intestinal efflux (Ai et al., 2021; Godos et al., 2024). Animal intervention dosages adopted in most preclinical rodent experiments are far higher than the clinically equivalent human doses calculated by body surface area conversion, making it difficult to extrapolate the *in vitro* and *in vivo* anti-pyroptotic effects observed in laboratory animals to human cardiovascular treatment. For glycosylated natural products including icariin and polydatin, intestinal microbiota deglycosylation further reduces the proportion of prototype drugs entering systemic circulation (Zarneshan et al., 2025; Zhao and Zhang, 2025). Polysaccharide metabolites such as Aconitum polysaccharide cannot efficiently penetrate the intestinal epithelial barrier via passive diffusion, resulting in negligible blood drug concentrations after oral delivery (Tian et al., 2022).

To tackle the above-mentioned pharmacokinetic limitations of single plant-derived metabolites, two mainstream strategies including structural chemical modification (Li et al., 2019) and cardiac-targeted lipid and polymer nanocarrier delivery systems (Li D. et al., 2024) have been fully validated in multiple preclinical cardiovascular studies. Such approaches simultaneously enhance aqueous solubility, elevate metabolic stability against intestinal and hepatic biotransformation, and facilitate specific enrichment of active compounds in myocardial tissue (Li D. et al., 2024).

#### 4.6.2 Ambiguous material basis of multi-metabolites multi-botanical botanical formulations

Unlike single plant-derived metabolites with definite chemical structures, multi-botanical botanical formulations rely on synergistic regulation of dozens to hundreds of metabolites to exert cardiovascular protective effects, which brings prominent research bottlenecks. At present, standardized extraction, separation and quality control technologies for most classical compound preparations are immature, and clear dose-effect relationships between core active metabolites and anti-pyroptotic phenotypes have not been established. It remains challenging to distinguish which metabolites contribute to the inhibition of NLRP3, caspase-4/5 or GSDME-mediated pyroptosis within a single compound formula. Integrated strategies combining network pharmacology (Zhang et al., 2023a), multi-omics and mass spectrometry metabolomics are widely adopted to screen core effective substances and interpret multi-target synergistic mechanisms of multi-botanical botanical formulations (Zhu et al., 2022), yet all such *in silico* predictive results merely generate preliminary research hypotheses and require independent *in vitro* and *in vivo* functional verification before drawing mechanistic conclusions.

Based on the above-mentioned material-basis challenges, the three subgroups of phytomedicines show distinct translational prospects. Purified monomers with well-defined chemical structures hold the greatest theoretical potential to evolve into pharmacological lead candidates, as they support target validation, structural modification and tailored pharmacokinetic optimisation. Nevertheless, most candidate monomers suffer from well-recognised limitations such as low oral bioavailability and rapid metabolic clearance, which must be overcome before clinical advancement. Defined extracts are appropriate primarily for preliminary activity screening; their complex constituent profiles, risk of PAINS-related assay artefacts, and difficulties in batch-to-batch standardisation impede their direct development into clinical drug entities. Compound prescriptions are valuable research tools for exploring multi-component synergistic mechanisms, yet substantial barriers remain for conventional small-molecule drug pipelines, including ambiguous core active ingredients, variable raw-material quality, and challenges in target deconvolution.

#### 4.6.3 Insufficient human clinical evidence and limitations of preclinical models

The regulatory effects of all natural agents summarized in this chapter are almost exclusively supported by *in vitro* cell culture and small rodent disease models, while large-sample, randomized, double-blind human clinical trials targeting pyroptosis-related cardiovascular endpoints are extremely scarce. Rodent atherosclerosis, diabetic cardiomyopathy and doxorubicin cardiotoxicity models cannot fully recapitulate human pathological microenvironments including hemodynamic characteristics, lipid metabolic profiles and inflammatory cell compositions. Human induced pluripotent stem cell-derived vascularized cardiac organoids can reconstruct multicardiac cell metabolites and recapitulate drug-induced cardiac injury phenotypes *in vitro*, which provides a humanized platform to simulate human-specific pyroptosis activation characteristics (Yang et al., 2024). It is critical to emphasize the editorial comment that relying solely on *in vitro* results to propose polyphenols as therapeutic CVD agents is largely implausible. However, the complex culture protocols and high experimental costs of cardiac organoids limit their large-scale application in high-throughput screening of natural small-molecule compounds.

It is necessary to address the risk of pan-assay interference compounds (PAINS) for the plant-derived metabolites summarized in this review. Flavonoids, alkaloids and polyphenols such as berberine, curcumin and myricetin all contain redox-active structural motifs that frequently generate false positive signals in pure *in vitro* cellular assays by non-specifically quenching fluorescence or chelating metal ions, which may overestimate their specific anti-pyroptotic activity (Bolz et al., 2021). Such off-target interference artifacts limit the independent translational value of single *in vitro* results. Nevertheless, the cardioprotective and pyroptosis-inhibitory effects of these metabolites have been repeatedly validated in multiple rodent cardiovascular disease models, which exclude the possibility that their bioactivity merely originates from assay interference. Additionally, multi-botanical botanical formulations contain diversified synergistic metabolites, which effectively reduce the single-molecule PAINS risk compared with isolated pure compounds. At present, standardized PAINS risk assessment systems are rarely adopted in preclinical botanical research, representing an important translational bottleneck restricting the clinical extrapolation of *in vitro* findings.

We systematically evaluate preclinical animal experimental schemes in accordance with the 4R (Reduce, Refine, Replace, Responsibility) ethical framework for animal research. Most relevant studies solely employ rodent models without complementary humanized research platforms, which results in excessive animal consumption and limited translational performance. Human induced pluripotent stem cell-derived cardiac organoids can recapitulate human-specific pyroptotic signaling, offering a superior alternative to lower animal usage and enhance the reliability of mechanistic exploration. Besides, many available animal studies only apply a single intervention dose rather than setting multiple concentration gradients, which restricts the comprehensive interpretation of dose-effect relationships.

#### 4.6.4 Uneven research depth of pyroptosis subtypes

Current mechanistic investigations of natural agents overwhelmingly focus on the canonical NLRP3/caspase-1/GSDMD pathway, while regulatory effects on non-canonical caspase-4/5 signaling and GSDME-dependent cardiomyocyte pyroptosis are relatively fragmentary and lack systematic elaboration. Few studies have adopted high-throughput structural simulation tools such as AlphaFold3 to predict the direct binding pockets between natural small molecules and gasdermin family proteins or inflammasome metabolites, leading to ambiguous clarification of direct vs. indirect regulatory modes for most compounds. All computational binding predictions are only hypothesis-generating and require wet-lab experimental confirmation to verify direct molecular interaction. More systematic research comparing compound activities across canonical, non-canonical and GSDME pyroptosis pathways is necessary to screen highly selective anti-cardiovascular injury lead compounds.

## 5 Conclusions and prospects

### 5.1 Overall research summary

This review systematically combed the core molecular mechanisms of canonical and non-canonical pyroptosis pathways, as well as the differential pyroptosis activation characteristics of cardiomyocytes, vascular smooth muscle, endothelial cells and cardiac fibroblasts under various cardiovascular pathological stimuli. Further, we summarized distinct preventive and therapeutic anti-pyroptotic regulatory mechanisms of single plant-derived metabolites and multi-botanical botanical formulations targeting pyroptosis in atherosclerosis, myocardial ischemia-reperfusion injury, diabetic cardiomyopathy, doxorubicin cardiotoxicity, heart failure and other cardiovascular disorders.

Existing preclinical data uniformly confirmed that natural active metabolites and compound prescriptions exert multi-target anti-inflammatory and cardioprotective effects by inhibiting NLRP3/caspase-1, caspase-4/5 and GSDME-mediated pyroptosis cascades. Compared with single-target chemical inhibitors, plant-derived agents exhibit unique advantages in intervening chronic complex cardiovascular injuries, due to their multi-metabolite synergistic regulatory properties. Nevertheless, the overall research system still has obvious limitations at the disciplinary level.

### 5.2 Integrated global research limitations and future research directions

Integrating all translational and methodological bottlenecks summarized in Section 4.6 and each subsection’s critical evaluations, we propose targeted, standardized research priorities fully aligned with Frontiers’ Four Pillars and ConPhyMP best-practice guidelines: standardize full botanical taxonomic validation, botanical ratio disclosure and extract quality control per POWO/MPNS and ConPhyMP norms; build uniform evaluation frameworks to assess PAINS false-positive risks for common polyphenol/alkaloid phytochemicals; set multi-gradient dosing regimens and restrict crude botanical extract *in vivo* doses below the 1 g/kg/day threshold while adopting human cardiac organoids to comply with the 4R animal ethics principle; conduct parallel comparisons across canonical, non-canonical and GSDME-dependent pyroptosis pathways to screen highly selective cardioprotective phytomedicines; and launch well-designed clinical trials with circulating pyroptosis biomarkers as primary clinical endpoints.

Most included preclinical papers only investigate the canonical NLRP3 inflammasome cascade, with insufficient systematic exploration of caspase-4/5-mediated non-canonical pyroptosis and GSDME-driven cardiomyocyte injury. Comparative assays across all three pyroptotic subtypes are rarely implemented, making it difficult to distinguish the subtype-specific activity of single phytochemicals and multi-botanical formulations, which hinders the discovery of potent, cardiac-selective lead agents. Future studies should perform parallel functional validation covering all three pyroptosis axes to identify high-efficiency, low-toxicity candidates that specifically mitigate cardiomyocyte pyroptosis.

Nearly all available mechanistic evidence merely relies on *in vitro* cell tests and rodent animal models. Large-scale, randomized controlled human trials using pyroptosis-related markers as primary outcomes remain extremely scarce, and universally recognized circulating cardiac pyroptosis biomarkers for clinical risk stratification and therapeutic efficacy assessment have not yet been established. Accordingly, subsequent research should prioritize screening and clinical validation of specific pyroptosis biomarkers, and design standardized clinical studies to quantify the correlation between pyroptosis levels and cardiac functional impairment in CVD patients.

Furthermore, most published work only carries out static single-pathway detection without integrating multi-omics, single-cell RNA sequencing and AI structural simulation to dissect the crosstalk between pyroptosis and other regulated cell death modalities such as ferroptosis and apoptosis. Pure *in silico* network pharmacology or molecular docking outputs cannot stand as conclusive mechanistic evidence without wet-lab functional validation, per journal requirements. Multi-omics profiling combined with AI-aided protein binding prediction tools like AlphaFold3 should be adopted to systematically map core regulatory networks of natural products and clarify the crosstalk among multiple cell death programs during pathological cardiac remodeling. AlphaFold-based structural simulation enables accurate prediction of direct molecular binding interactions between phytochemicals and target proteins, providing solid guidance for subsequent functional verification experiments (Abramson et al., 2024).

### 5.3 Concluding remarks

Pyroptosis acts as a core inflammatory driver across nearly all common cardiovascular diseases. Abundant preclinical data show plant-derived metabolites widely distributed across global medicinal flora, not exclusive to Chinese botanical drugs, and multi-botanical botanical formulations recorded in Chinese pharmacopoeia have promising preventive anti-pyroptotic prospects. However, polyphenolic compounds lack reliable therapeutic efficacy for diagnosed cardiovascular lesions if only supported by *in vitro* evidence and further tiered translational research combining humanized models and clinical cohorts is required to break current bottlenecks.
